# Supplementary material for: Genome-wide effects of the antimicrobial peptide apidaecin on translation termination in bacteria
Source: eLife. 2020 Oct 8;9:e62655. doi: 10.7554/eLife.62655 (PMC7544508; doi:10.7554/eLife.62655)
Supplement: Source data 7. [file elife-62655-data7.docx]

**Source Data 7. Sensitivity towards Api of cells lacking ribosome rescue systems.**

MIC values of Api against *E. coli* BW25113 cells lacking individual ribosome rescue systems. The SmpB protein is essential for the operation of the tmRNA-based ribosome rescue system (Buskirk and Green, 2017). All the tested strains were acquired from the Keio collection (Baba et al., 2006) and their identity was verified by PCR. Note that a two-fold difference in MIC is considered to be within the experimental error.

| ***E. coli* strain** | **Api MIC (µM)** |
| --- | --- |
| BW25113 (wt) | 12.5 |
| BW25113 Δ *arfA* | 6.25 |
| BW25113 Δ *arfB* | 12.5 |
| BW25113 Δ smpB | 25 |
