# Supplementary material for: Genome-wide effects of the antimicrobial peptide apidaecin on translation termination in bacteria
Source: eLife. 2020 Oct 8;9:e62655. doi: 10.7554/eLife.62655 (PMC7544508; doi:10.7554/eLife.62655)
Supplement: Transparent reporting form [file elife-62655-transrepform.docx]

***eLife’s* transparent reporting form**

We encourage authors to provide detailed information *within their submission* to facilitate the interpretation and replication of experiments. Authors can upload supporting documentation to indicate the use of appropriate reporting guidelines for health-related research (see [EQUATOR Network](http://www.equator-network.org/%20)), life science research (see the [BioSharing Information Resource](https://biosharing.org/)), or the [ARRIVE guidelines](http://www.plosbiology.org/article/info:doi/10.1371/journal.pbio.1000412) for reporting work involving animal research. Where applicable, authors should refer to any relevant reporting standards documents in this form.

If you have any questions, please consult our Journal Policies and/or contact us: [editorial@elifesciences.org](mailto:editorial@elifesciences.org).

**Sample-size estimation**

- You should state whether an appropriate sample size was computed when the study was being designed
- You should state the statistical method of sample size computation and any required assumptions
- If no explicit power analysis was used, you should describe how you decided what sample (replicate) size (number) to use

Please outline where this information can be found within the submission (e.g., sections or figure legends), or explain why this information doesn’t apply to your submission:

No explicit power analysis was used for Ribo-seq experiment. Instead, we followed the customary and widely-accepted practice of running each sample in two biological replicates. For the proteomics identification of extended peptide in each sample, statistical test was performed using reversed sequences in the reference database to estimate the false discovery rate at spectrum, peptide and protein level. For protein pathway enrichment analysis, 12 measurements were used for each sample and the false discovery rate was calculated for each individual pathway identified.

**Replicates**

- You should report how often each experiment was performed
- You should include a definition of biological versus technical replication
- The data obtained should be provided and sufficient information should be provided to indicate the number of independent biological and/or technical replicates
- If you encountered any outliers, you should describe how these were handled
- Criteria for exclusion/inclusion of data should be clearly stated
- High-throughput sequence data should be uploaded before submission, with a private link for reviewers provided (these are available from both GEO and ArrayExpress)

Please outline where this information can be found within the submission (e.g., sections or figure legends), or explain why this information doesn’t apply to your submission:

In biological replicates used in Ribo-seq experiment, each sample was grown as an independent culture and processed independently from each other up to the linker ligation step of the Ribo-Seq library preparation protocol (McGlincy and Ingolia, 2017).

The outliers for the box graph shown in Fig. 3B were discarded using the automated function ROUT (Q = 1%) of Prism (GraphPad) software package.

For proteomics data analysis, protein, peptide and spectrum identifications were all controlled at < 1% false discovery rate. For pathway enrichment analysis, only highly confident peptides with multiple peptide spectrum matches were selected. All proteins identified with less than 5 peptide spectrum count was excluded from pathway enrichment analysis.

The RNA-Seq and Ribo-Seq data can be found in NCBI Gene Expression Omnibus (GEO) database under accession number GSE150034 with the reviewer token wjqtuosqhvstvsj

The proteomics data can be found in the EMBL-EBI Proteomics Identification database (PRIDE) using the following credentials: username reviewer13830@ebi.ac.uk, password: e7p7jdNS

**Statistical reporting**

- Statistical analysis methods should be described and justified
- Raw data should be presented in figures whenever informative to do so (typically when N per group is less than 10)
- For each experiment, you should identify the statistical tests used, exact values of N, definitions of center, methods of multiple test correction, and dispersion and precision measures (e.g., mean, median, SD, SEM, confidence intervals; and, for the major substantive results, a measure of effect size (e.g., Pearson's r, Cohen's d)
- Report exact p-values wherever possible alongside the summary statistics and 95% confidence intervals. These should be reported for all key questions and not only when the p-value is less than 0.05.

Please outline where this information can be found within the submission (e.g., sections or figure legends), or explain why this information doesn’t apply to your submission:

The statistical information can be found in the figure legends and in Materials and Methods section.

(For large datasets, or papers with a very large number of statistical tests, you may upload a single table file with tests, Ns, etc., with reference to sections in the manuscript.)

**Group allocation**

- Indicate how samples were allocated into experimental groups (in the case of clinical studies, please specify allocation to treatment method); if randomization was used, please also state if restricted randomization was applied
- Indicate if masking was used during group allocation, data collection and/or data analysis

Please outline where this information can be found within the submission (e.g., sections or figure legends), or explain why this information doesn’t apply to your submission:

Not applicable to this work

**Additional data files (“source data”)**

- We encourage you to upload relevant additional data files, such as numerical data that are represented as a graph in a figure, or as a summary table
- Where provided, these should be in the most useful format, and they can be uploaded as “Source data” files linked to a main figure or table
- Include model definition files including the full list of parameters used
- Include code used for data analysis (e.g., R, MatLab)
- Avoid stating that data files are “available upon request”

Please indicate the figures or tables for which source data files have been provided:

The relevant additional data files are provided with the manuscript.
